# Evaluation of thymol application for anaesthesia of adult zebrafish

**DOI:** 10.1007/s10695-025-01574-z

**Published:** 2025-09-01

**Authors:** Luís Félix, Sandra M. Monteiro, Carlos Venâncio

**Affiliations:** 1 Centre for the Research and Technology of Agroenvironmental and Biological Sciences, CITAB, Inov4Agro, Universidadede Trás-Os-Montes E Alto Douro, UTAD, Quinta de Prados, 5000-801 Vila Real, Portugal; 2 Animal and Veterinary Research Centre (CECAV), UTAD, Quinta de Prados, 5000-801 Vila Real, Portugal

**Keywords:** Anaesthesia, Aquaculture, Monoterpenes, Plant-derived anaesthetics, Sedation, Welfare, Zebrafish

## Abstract

The use of anaesthetics has become extremely important in promoting responsible welfare practices in aquaculture and research. However, adverse reactions and side effects have been described for commonly used anaesthetics such as MS-222 and eugenol. This study examined thymol as a new anaesthetic for adult zebrafish (*Danio rerio*), a species for which no previous thymol anaesthesia studies have been reported. Concentration–response tests (25–200 mg/L) were conducted using 10 individual animals per concentration, along with assessments of cardiorespiratory, locomotion, and aversive-related effects. Concentrations below 25 mg/L did not induce anaesthetic effects while 50 and 75 mg/L were most effective, inducing deep anaesthesia with average induction times of around 300 s and recovery times of between 400 and 500 s. Induction times at these concentrations were comparable to those observed with MS-222 and eugenol; however, recovery periods were longer. No mortality was observed at these concentrations, although transient cardiorespiratory depression was noted. Video tracking analyses revealed no significant behavioural changes at the lowest concentrations. In contrast, concentrations above 150 mg/L showed narrow behavioural safety margins and induced aversive responses, rendering them unsuitable for fish aesthesia. Based on these findings, a short 10-min exposure to 50 mg/L thymol resulted in cortisol levels comparable to eugenol and lower than in the control group. These findings support the use of 50 mg/L thymol as an effective and welfare-conscious anaesthetic for adult zebrafish. However, further research is recommended to elucidate thymol’s physiological effects and its mechanism of action in this species.

**Supplementary Information:**

The online version contains supplementary material available at 10.1007/s10695-025-01574-z.

## Introduction

The global consumption of farmed fish and seafood has seen a notable increase in recent years and is expected to continue growing (Naylor et al. 2021). Concurrently to the growth of the aquaculture industry (Garlock et al. 2019; Naylor et al. 2021), there has been an increased use of aquatic animals in ecotoxicology, genetic, and biomedical-related research areas (Pouil et al. 2020; Mocho and von Krogh 2022; Supriya et al. 2022). However, in recent years, a growing body of scientific evidence has confirmed that fish are sentient animals, capable of experiencing pain, stress and distress similar to other vertebrate species (Lambert et al. 2022). Although this understanding is now widely recognized in the scientific community, the broad adoption of animal welfare principles and ethical practices in aquaculture and research is still evolving (Browman et al. 2019; Toni et al. 2019; Seibel et al. 2020; Browning 2023). Nonetheless, awareness has been increasing, particularly as poor welfare practices have been associated with major economic consequences (Toni et al. 2019). One of the primary strategies to improve welfare across aquaculture and laboratory protocols is the use of anaesthetics (Sloman et al. 2019; Schroeder et al. 2021).

The most used anaesthetic for fish in aquaculture and research activities is ethyl 3-aminobenzoate methanesulfonate, also known as tricaine methanesulfonate (MS-222) (Carter et al. 2011; Topic Popovic et al. 2012; Lidster et al. 2017). However, the reported response inconsistencies (Readman et al. 2017) and concentration- and species-dependent side effects (Carter et al. 2011) have prompted the search for potential safer alternatives. This has involved researching plant-derived essential oils and their bioactive compounds (Tsuchiya 2017; Hoseini et al. 2019; Souza et al. 2019; Aydın and Barbas 2020). In this context, eugenol, which comprises around 80% of clove oil (Javahery et al. 2012; Fernandes et al. 2017), has been identified as a potential anaesthetic for different fish species, primarily acting as a modulator of GABA_A_ receptors (Lehotzky et al. 2023). Nonetheless, reports have emerged regarding potential seizure-inducing activity (Barbas et al. 2021) and severe lesions (Ayala-Soldado et al. 2023). Given these limitations, attention has turned to less-studied compounds in the monoterpene family many of which have been noted for their anaesthetic properties (Félix et al. 2023), attributed to their chemical resemblance to local anaesthetics (Tsuchiya 2017). Among them, thymol is known to modulate the central nervous system by enhancing GABAergic neurotransmission which is believed to underlie its sedative, anxiolytic, and anaesthetic effects (Priestley et al. 2003; Garcia et al. 2006), making it a promising candidate for fish anaesthesia. Experimental studies in different fish species have shown thymol’s anaesthetic and/or sedative potential (Bianchini et al. 2017; Yousefi et al. 2018, 2022; Aydın and Orhan 2021; Boaventura et al. 2022). However, to date, the study of thymol as an alternative anaesthetic agent for adult zebrafish (*Danio rerio*) has not been explored, although its analgesic and anaesthetic profiles have been recently outlined for larval stages of this species (Rocha et al. 2024; Vieira et al. 2024). This tropical teleost fish has emerged as a superb vertebrate model not just for scientific research (Cassar et al. 2020; Choi et al. 2021) but also with promising prospects for aquaculture application (Ulloa et al. 2014; Lee-Estevez et al. 2018; Jorgensen 2020; Piferrer and Ribas 2020). Hence, this study aimed to evaluate the physiological, behavioural, and cardiorespiratory responses in zebrafish anesthetized with thymol and compare these responses with those induced by MS-222 and eugenol. The hypothesis posited that zebrafish anesthetized with thymol would exhibit induction and recovery times, behavioural responses, and physiological stress markers comparable to those observed with the most commonly used anaesthetics (MS-222 and eugenol), thus facilitating the exploration of thymol as an effective and alternative anaesthetic for this species.

## Methods

### Chemicals

Thymol (99%, CAS 89–83-8) and MS-222 (ethyl 3-aminobenzoate methanesulfonate, 98%, CAS 886–86-2) were purchased from Merck (Algés, Portugal) while eugenol (99%, CAS 97–53-0) was obtained from Alfa Aesar (Kandel, Germany). A stock solution of 89 g L^−1^ (thymol and eugenol, pH 7.18 ± 0.02 and 7.09 ± 0.08, respectively) was prepared in 90% ethanol, while a stock solution of 1500 mg L^−1^ (MS-222) was prepared in distilled water and neutralized to pH 7.2–7.4 with sodium bicarbonate. All solutions were stored at 4 ºC until further dilution.

### Animals and housing

**Fig. 1:**
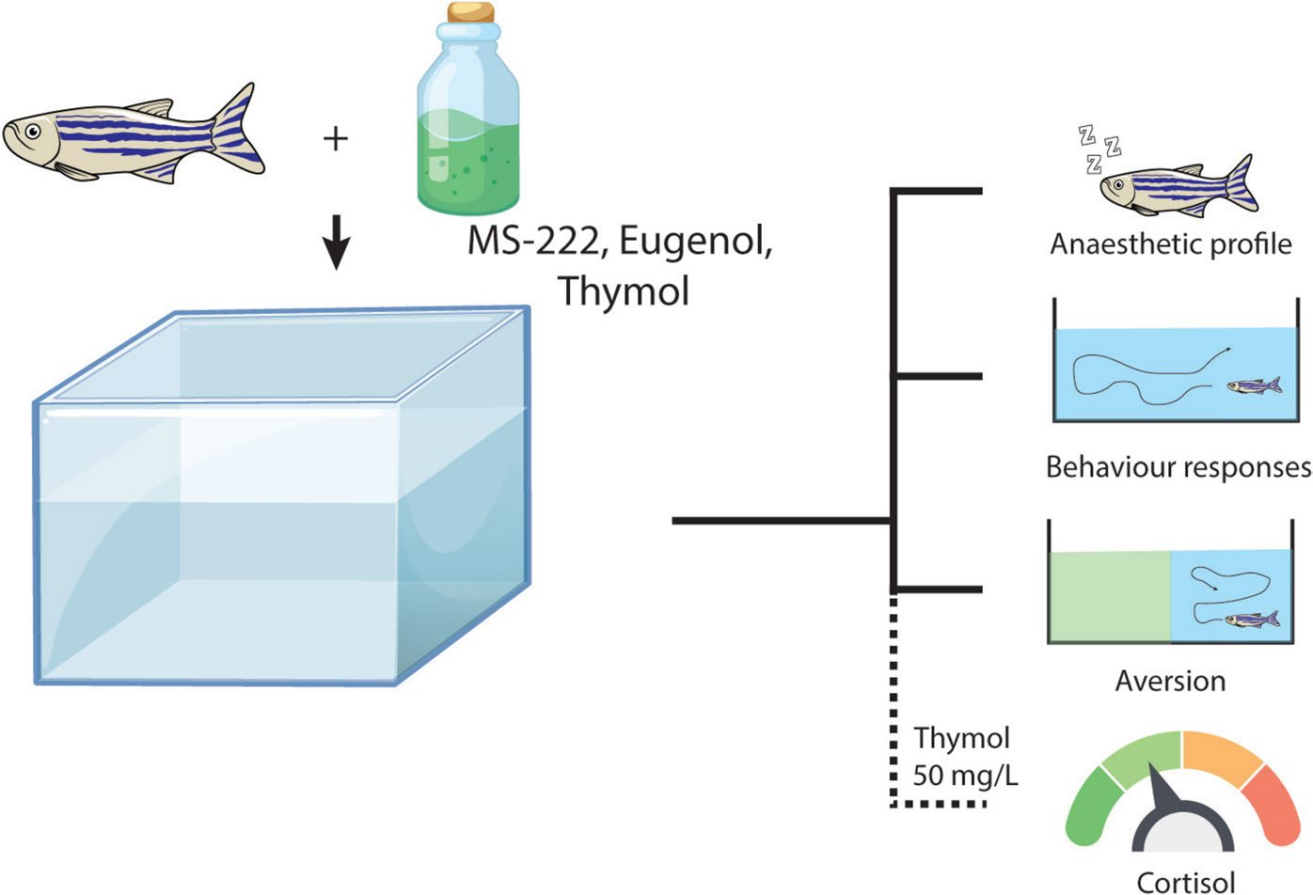
Diagram of the experimental design and analysed endpoints. Adult zebrafish were individually and randomly assigned to MS-222, eugenol, or varying concentrations of thymol to evaluate their anaesthetic profile, locomotion, and behavioural aversion. Based on these results, a 10-min exposure to a 50 mg/L thymol concentration was conducted to assess potential stress-related alterations

Adult AB zebrafish (*Danio rerio*) were reared from hatching and kept at a density of 2–3 fish per litre (Andersson and Kettunen 2021) in the animal facilities of the University of Trás-os-Montes and Alto Douro (Vila Real, Portugal), following standard protocols. The 20-L glass tanks were supplied through an open system with aerated, dechlorinated, charcoal-filtered, and UV-sterilized tap water from the City of Vila Real. They were maintained as outlined: dissolved oxygen, 7.8 ± 0.7 mg/L; pH, 7.2 ± 0.3; temperature, 28.3 ± 0.5 °C; conductivity, 708 ± 128 μS/cm; alkalinity, 28.0 ± 10.2 mg/L as CaCO_3_; total hardness, 42.2 ± 13.6 mg/L as CaCO_3_; total ammonia nitrogen, 0.2 ± 0.2 mg/L; unionized ammonia, 0.0 ± 0.0 mg/L; nitrite, 0.1 ± 0.1 mg/L; and nitrate, 5.9 ± 5.1 mg/L. The fish tanks followed a 14-h light and 10-h dark cycle, and the animals were fed a nutritionally balanced diet (Zebrafeed, Sparos Lda., Portugal) twice daily. Fish of both sexes, aged between 12 to 18 months post-hatching, with an average body weight of 0.36 ± 0.12 g and body length of 3.38 ± 0.34 cm, were selected for the experiments. This choice was made considering that no gender-specific effects of anaesthesia have been reported in zebrafish for eugenol and MS-222 (Musk et al. 2020), while no information could be found in the literature regarding thymol. Before experiments, the animals underwent a 24-h fasting period to minimise the potential for regurgitation and the excretion of nitrogenous waste (Neiffer and Stamper 2009). Animals were handled following the standard protocols outlined by the European Animal Welfare Directive 2010/63/EU and Portuguese Legislation (Decreto-Lei 113/2013 changed by Decreto-Lei n.º 1/2019). Furthermore, the experimental protocol underwent ethical review and approval by the ORBEA (Orgão Responsável pelo Bem-Estar dos Animais/Animal Welfare Body, 1805-e-CITAB-2023) and the CE-UTAD (Ethics Committee, Doc49-CE-UTAD-2023) of the University of Trás-os-Montes and Alto Douro, as well as by the DGAV (Direção Geral de Alimentação e Veterinária/Directorate General for Food and Veterinary, 37,744/25-S). A depiction of the experimental procedure employed in this study can be found in Fig. 1.

### Anaesthetic profile

One hundred fish were utilized to assess the induction and recovery times from thymol anaesthesia which were taken from the stock tank and transferred to the experimental acrylic containers (7.6 × 9.5 × 8.3 cm) consisting of 200 mL of the test concentrations. These varied from 25 to 200 mg/L (25, 50, 75, 100, 150 and 200 mg/L) while MS-222 (150 mg/L, (Collymore et al. 2014)) and eugenol (80 mg/L, (Grush et al. 2004)) were selected according to the available literature. Two control groups were observed under identical conditions: one exposed to anaesthetic/ethanol-free water and the other to the maximum concentration of ethanol required to dilute the highest concentration of thymol (0.2%) (Can et al. 2019). Fresh anaesthetic baths were provided for each animal, and the treatment order was determined using an online randomization tool (randomization.com). No blind approach was implemented due to the distinctive odour of the natural compounds. The fish were individually monitored (n = 10 for each concentration) by the same observer until stage 3 anaesthesia became evident (Sneddon 2012; Barbas et al. 2016). In brief, the duration until the loss of equilibrium and erratic swimming despite maintaining responsiveness to pressure on the caudal peduncle (stage A2), as well as the complete loss of reflex activity and responsiveness to external stimuli (stage A3), were documented using a digital stopwatch. Each stage had a maximum observation time of 10 min, after which the trial was concluded to prevent undue stress on the fish. Upon reaching stage A3, the animals were removed from the anaesthetic bath and promptly transferred to a separate plastic container filled with an equal volume of clean water. The time taken to regain equilibrium (stage R1) and to fully re-establish a normal swimming pattern (stage R2) were also noted. The recovery assessment was not time-constrained but the cardiorespiratory function was evaluated after a certain time as described below. Following complete recovery, the animals were returned to their maintenance tanks and observed for 96 h to monitor for potential mortality and behavioural changes. They were only reused for further experimentation if no noticeable morphological or behavioural alterations were observed, thereby minimising overall animal usage.

### Cardiac and respiratory signals

Following the induction of stage 3 anaesthesia, the fish were promptly relocated to a glass petri dish filled with the identical anaesthetic solution, alongside a weighted sponge equipped with a groove to securely position the fish, ensuring its ventral side faced upward. A SZX7 stereomicroscope (Olympus, Tokyo, Japan) paired with an EP50 digital camera (Olympus, Tokyo, Japan) was employed for observation in a room maintained at 27–28 ºC. The control animals underwent a 5-min sedation period using 50 mg/L of MS-222 before analysis (Le et al. 2019b). After a 30-s acclimation period, a 1-min video was captured utilising cold light illumination (Mousavi and Patil 2020). Subsequently, the heart rate was assessed through a direct count of the recorded video (Pylatiuk et al. 2014). The respiratory rate was also determined through visual inspection of the recorded videos (Martins et al. 2018). When required, the video speed was adjusted using the open-source software VLC Media Player (version 3.0.20, VideoLAN, Paris, France).

### Behavioural responses to anaesthesia

To assess behavioural responses during anaesthesia induction with thymol, a 3D video recording system was utilised, as detailed in another source (Audira et al. 2018). A pool of 100 randomly selected adult animals was employed for this experiment, and they were reused if no noticeable morphological or behavioural alterations were observed within one week of exposure. Briefly, an acrylic box measuring 10 × 10 × 10 cm was enveloped with non-reflective white sticker paper, leaving only the front face uncovered to minimize distractions. A mirror was affixed to the box lid at an angle allowing observation of the entire bottom of the box. Each animal (n = 10 per group) was individually placed into this enclosure, which held 400 mL of the described anaesthetic concentrations. Immediately afterwards, high-definition videos lasting 10 min were captured using a smartphone camera (resolution: 1920 × 1080 pixels/30 frames per second) positioned in front of the tank. The 3D video recordings were subsequently analysed using TheRealFishTracker automated tracking system (Buske and Gerlai 2014) to capture XY and YZ coordinates for each fish. Behavioural parameters including total distance travelled, average speed, meandering (undirected movement), and freezing duration were derived from the coordinates (Audira et al. 2018), as outlined previously, during the initial 2 min or until the average time at which the animal lost equilibrium. In addition, 3D swimming trajectory plots were obtained from the data by using Origin software from OriginLab (Northampton, USA) as outlined elsewhere (Audira et al. 2018). The immobility (speed less than 1 cm/s), swimming (speed between 1 and 10 cm/s), and rapid movement (speed exceeding 10 cm/s) of zebrafish were evaluated based on the 3D plots, following the described methodology.

### Preference evaluation

To assess the possible discomfort associated with thymol anaesthesia, a two-chamber preference test setup was built (Abreu et al. 2016; Junior et al. 2018), where fish were given the choice to choose between clean water and water treated with different thymol concentrations. In brief, a white acrylic tank measuring 18 × 26 × 8 cm, equipped with sponges and fine mesh, was utilised to create two separate compartments with unmixed laminar flows (Video S1), generated by two circulating pumps operating at a flow rate of 0.8 L/min. This flow rate was adjusted to ensure the laminar flow did not impose significant challenges or stress on the tested fish. An experimental area, measuring 18 × 10 × 8 cm, allowed fish to move freely between compartments. The water used was sourced from maintenance tanks, and 100 random fish (with 10 fish per concentration) were individually introduced into the experimental area. After an acclimation period of 150 s, various anaesthetic concentrations were introduced, except 200 mg/L due to the low recovery rates. Videos were recorded from above using a smartphone camera (1920 × 1080 pixels at 30 frames per second). The fish's location during the 150-s exposure period was analysed using ANY-maze tracking software (Stoelting Co., USA). These time intervals were chosen based on prior research that demonstrated fish exhibiting aversive reactions to anaesthetics (Readman et al. 2013). After the fish exposure, the system underwent manual flushing and thorough cleaning, with the anaesthetic compartment being switched to prevent any potential bias. Prior to conducting any assay, confirmation of unmixed laminar flow was ensured by using a non-toxic coloured food dye. Additionally, both a negative control (maintenance tank water) and a positive control utilising hydrochloric acid (pH 3.01 ± 0.05, as per (Readman et al. 2013)) were tested under identical conditions. After the experiment, the animals were reintroduced to the stock tank and were subsequently reused in subsequent experiments following a recovery period.

### Short anaesthesia and cortisol levels

Adult fish are frequently subjected to anaesthesia for procedures that typically necessitate brief periods of sedation (Sneddon 2012; Schroeder et al. 2021). Considering this and guided by the established anaesthetic properties of thymol, new experiments were conducted involving 50 randomly selected adult zebrafish subjected to longer anaesthesia durations (10 min). Initially, trials were carried out using the same concentrations of MS-222 (150 mg/L) and eugenol (80 mg/L), along with a non-aversive concentration of thymol (50 mg/L, as determined in the previous tests). However, due to the observed low survival rate of fish after 24 h of exposure to eugenol (0% survival), its concentration was reduced to 50 mg/L. Fish (10 fish per concentration) were monitored under conditions identical to those described to determine the anaesthetic profile of thymol. Individual fish were randomly placed in the acrylic tank, and upon reaching stage 3 anaesthesia, fish were maintained in the anaesthetic solution for 10 min. Subsequently, the animals were transferred to a glass petri dish containing a sponge soaked with the same anaesthetic solution, with a gap provided. The two control groups (maintenance water and 0.2% ethanol) were sedated using 50 mg/L MS-222, following the procedure outlined above. Although exposure to MS-222 can lead to alterations in cortisol levels shortly after exposure, this stress reaction is dependent upon the concentration, potentially varying among species (Thomas and Robertson 1991), and influenced by the duration of exposure (Welker et al. 2007). Despite a lack of specific studies in zebrafish, findings from other fish species suggest that MS-222 does not significantly increase the cortisol response until it reaches stage 3 anaesthesia (Crosby et al. 2006; Welker et al. 2007). The mucus was then collected using the two ends of sterile swabs as described (Jorge et al. 2023) and validated earlier (Jorge et al. 2024). Subsequently, the animals were permitted to recuperate and were reintroduced to the maintenance tank. The two ends of the swab were gathered in 500 μL ice-cold phosphate-buffered saline (PBS, pH 7.4) and then frozen at −20 ºC. To extract cortisol, 1 mL of diethyl ether was added to the sample and left to stand overnight, following the procedure outlined in previous publications (Laberge et al. 2019; Ferreira et al. 2022). After freezing, the organic layer was transferred to a new tube and evaporated using a speed vac (Labconco Centrivap 78,120–00) at 45 ºC. Subsequently, the dried samples were reconstituted in 100 μL of PBS and allowed to rest overnight. Cortisol levels were quantified using the DetectX Cortisol ELISA Kit (Arbor Assays, K003-H, Ann Arbor, USA) following the manufacturer’s instructions. Cortisol levels were measured at 405 nm with a correction at 490 nm and normalised by protein content, determined at 280 nm (Noble and Bailey 2009), using a microplate spectrophotometer (PowerWave XS2, Bio-Tek Instruments, Winooski, USA).

### Statistical analysis

A priori sample size was estimated based on findings from cited studies, assuming a two-tailed ANOVA for independent groups to detect a medium to large effect, with a power exceeding 0.8 at a significance level of 0.05. The Shapiro–Wilk and Brown-Forsythe tests were employed respectively to assess the normality of distribution and homogeneity of variance in the raw data before statistical analyses. One-way analysis of variance (ANOVA) was used to compare differences in normally distributed data, with the Tukey post hoc test utilised for multiple comparisons at a significance level of 0.05. In cases where the data did not conform to normal distribution, the non-parametric Kruskal–Wallis test was employed. To assess the aversive effects of anaesthetics the t-test was utilised. Statistical analysis was conducted using GraphPad Prism 9.1 software (GraphPad Software Inc., San Diego, USA).

## Results

### Thymol anaesthetic profile

**Fig. 2:**
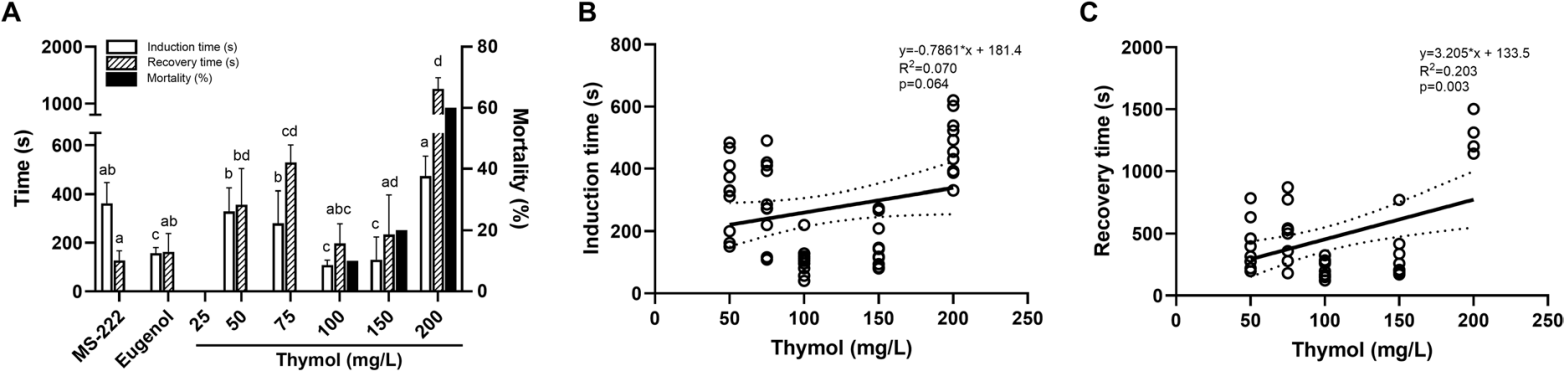
Induction and recovery times of fish subjected to varying thymol concentrations and the resulting mortalities **(A).** The values are presented as median and interquartile ranges from ten independent exposures. Different letters indicate significant differences between groups (p < 0.05). Relationship between induction time (**B**) or recovery time (**C**) and the different thymol concentrations in zebrafish. Linear regression lines and 95% confidence interval bands, with regression statistics, are presented. The results for the 25 mg/L concentration are not shown as no deep anaesthesia was obtained

The average durations for anaesthesia induction and recovery across various thymol concentrations are depicted in Fig. 2A. No sedative or anaesthetic effects were noted in either the fish subjected to ethanol exposure or the control fish. Similarly, fish exposed to 25 mg/L of thymol did not attain a state of profound anaesthesia, although sedative effects were evident (Supplementary Table 1). Consequently, these specific groups are omitted from Fig. 2A while increasing concentrations of thymol (50, 75, 100, 150, and 200 mg/L) resulted in different anaesthetic outcomes. In this regard, increasing concentrations of thymol caused a faster anaesthesia induction until 100 mg/L, at which point the curve inverted, resulting in an increased anaesthesia induction time. Given this, concentrations equal to or exceeding 100 mg/L induced elevated mortality rates post-anaesthesia, reaching 60% in fish anesthetised using the highest thymol concentration. Nevertheless, compared to MS-222, the 100 and 150 mg/L could induce a faster deep anaesthesia state (p = 0.0004 and p = 0.011, respectively). Compared to eugenol, fish exposed to 200 mg/L of thymol required more time to achieve a comparable depth of anaesthesia (p = 0.002), whereas no notable variances were detected at the remaining concentrations. Regarding the full recovery, no statistical difference was observed at concentrations of 100 and 150 mg/L when compared to MS-222 and eugenol while more time was needed to observe the complete recovery with lower concentrations (p < 0.05). In parallel, animals also took more time to recover from the highest thymol concentration tested (200 mg/L, p < 0.004). Based on these results, there was no significant correlation between thymol concentrations and induction time (R^2^ = 0.070, p = 0.064) but a positive correlation between thymol concentrations and recovery times (R^2^ = 0.203, p = 0.003) (Fig. 2B and C, respectively) was noted.

### Cardiac and respiratory effects of thymol

**Fig. 3:**
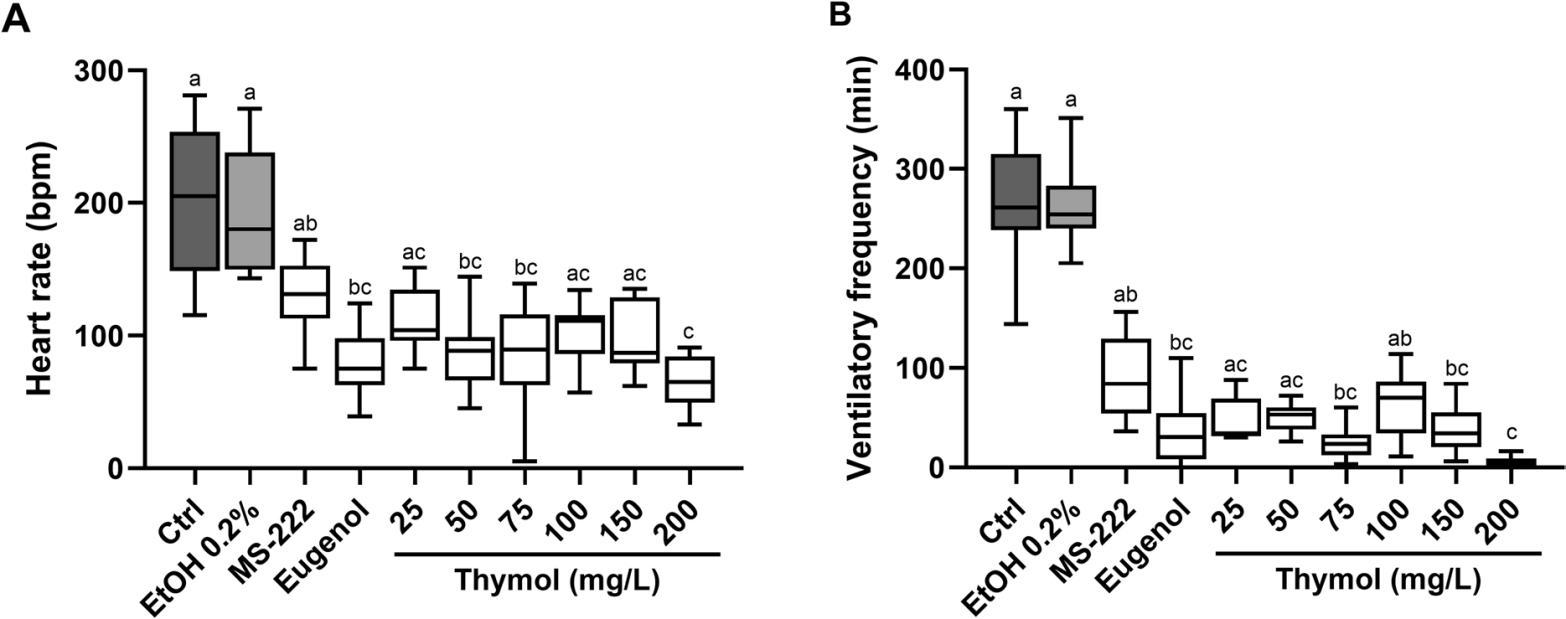
Effects of thymol anaesthesia on heart rate **(A)** and ventilatory frequency **(B)** of zebrafish. The values are presented as median and interquartile ranges from ten independent replicate exposures and were analysed by the Kruskall-Wallis test followed by Dunn’s post hoc test. Different letters indicate significant differences between groups (p < 0.05). bpm: beats per minute

Figure 3 illustrates the impact of anaesthesia with different concentrations of thymol on the heart rate and ventilatory frequency of adult zebrafish, with the raw data provided in Supplementary Table 1. Concerning the heart rate (Fig. 3A), no alteration was observed due to ethanol exposure compared to the control group. Regarding thymol exposure, 50, 75 and 200 mg/L caused a significant decrease in the heart rate compared to the control group (p < 0.05), while no significant changes were observed for 25, 100 and 150 mg/L, despite the reduction in heart rate. Compared to MS-222, which induced a decrease that was not statistically different from the control group, only the highest thymol concentration (200 mg/L) caused a significant decline in the heart rate (p = 0.009). No notable differences were observed between fish anaesthetised with thymol and those anaesthetised with eugenol. Concerning the respiratory rate (Fig. 3B), a depression was induced by all anaesthetics with varying depths. Thymol concentrations above 75 mg/L (except for 100 mg/L) statistically depressed the rate of respiratory movements against the control group but similar to eugenol. Conversely, lower concentrations of thymol (25 and 50 mg/L) and MS-222 caused a non-significant reduction in respiratory rate compared to the control group.

### Thymol effects on behavioural parameters

**Fig. 4:**
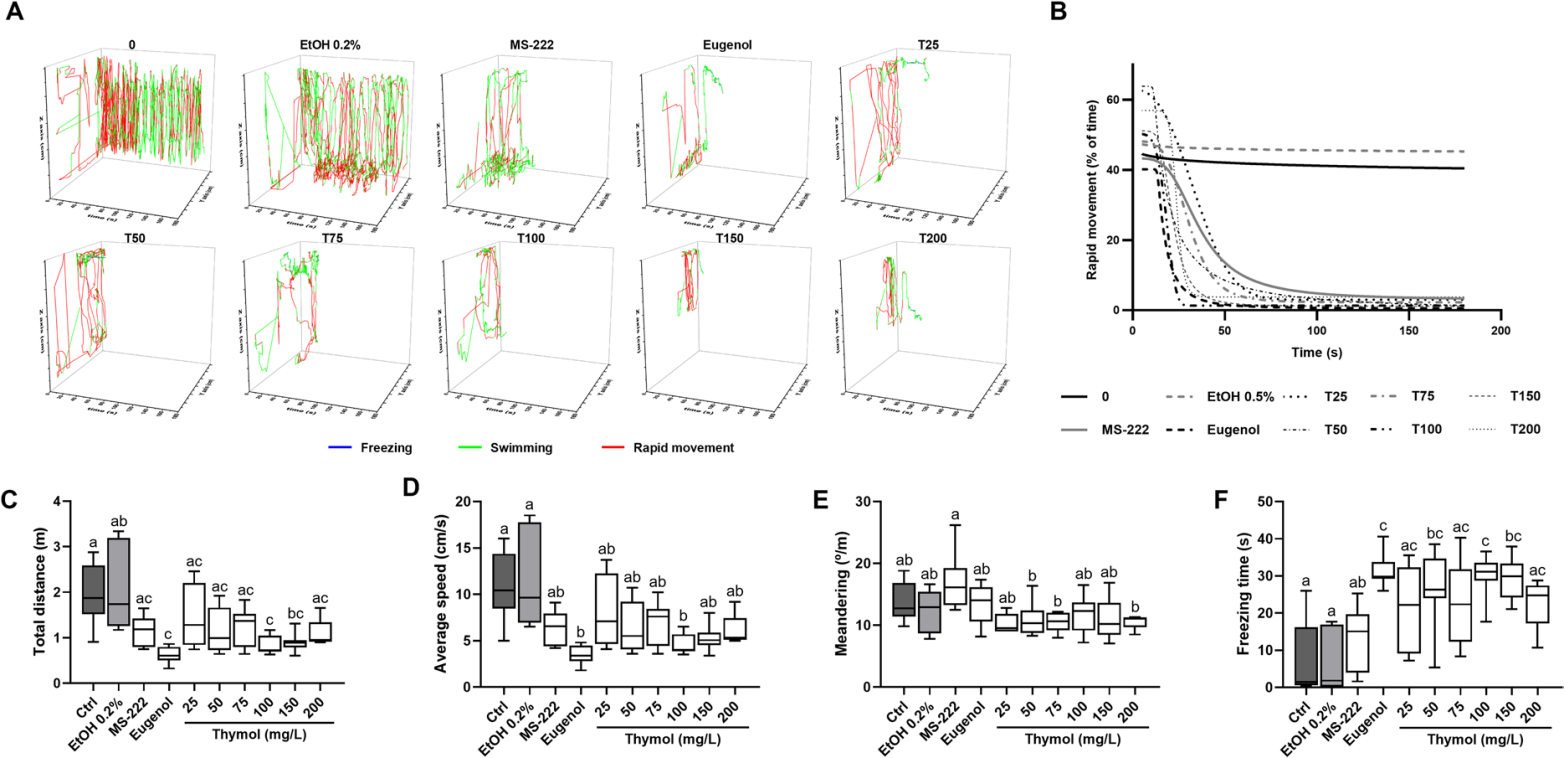
Representative three-dimensional swim trajectories of zebrafish exposed to thymol **(A**) and the percentage of time the fish exhibit rapid movement during anaesthesia induction (**B**). Locomotion profiles of zebrafish during induction: total distance travelled (**C**), average speed (**D**), meandering (**E**), and freezing time (**F**). The data are expressed as the median and interquartile ranges from ten independent anaesthesia inductions and were analysed by the Kruskall-Wallis test followed by Dunn’s post hoc test. Different letters indicate significant differences between groups (p < 0.05)

Figure 4 displays the behavioural characteristics evaluated during the induction of anaesthesia at various concentrations of thymol, with the original data provided in Supplementary Table 2. The temporal 3D reconstruction plots generated from the X, and Y-coordinates (depicted in Fig. 4A) revealed that animals anaesthetised with thymol initially exhibited hyperactivity, swimming both vertically and horizontally in the tank, for a brief period, before ceasing movement. However, after immobilisation, spasms and/or swimming in"corkscrew"circles were observed, particularly at high thymol concentrations. At lower concentrations (25 mg/L), the behaviour was similar to that observed in the control and ethanol groups while a concentration-dependent reduced kinematics was observed, as depicted in Fig. 4B. In terms of locomotion parameters, the data revealed a decrease in both total distance travelled and average speed (Fig. 4C and D, respectively) for animals exposed to 100 mg/L compared to the control and ethanol groups (p < 0.05). However, no significant alterations were observed for the other concentrations of thymol, despite the variations detected. Still, data obtained for 100 mg/L was statistically similar to that observed in the eugenol group. There were no notable alterations for meandering (Fig. 4E) when compared to the control groups, and only slight variations were perceived concerning the MS-222 group. Similarly, a significant freezing time was observed for 100 mg/L in relation to the control group and MS-222 (p < 0.05), which was not different from the eugenol group. For the remaining thymol concentrations, fluctuations were noticed with thymol inducing an increase in freezing time.

### Preference test

**Fig. 5:**
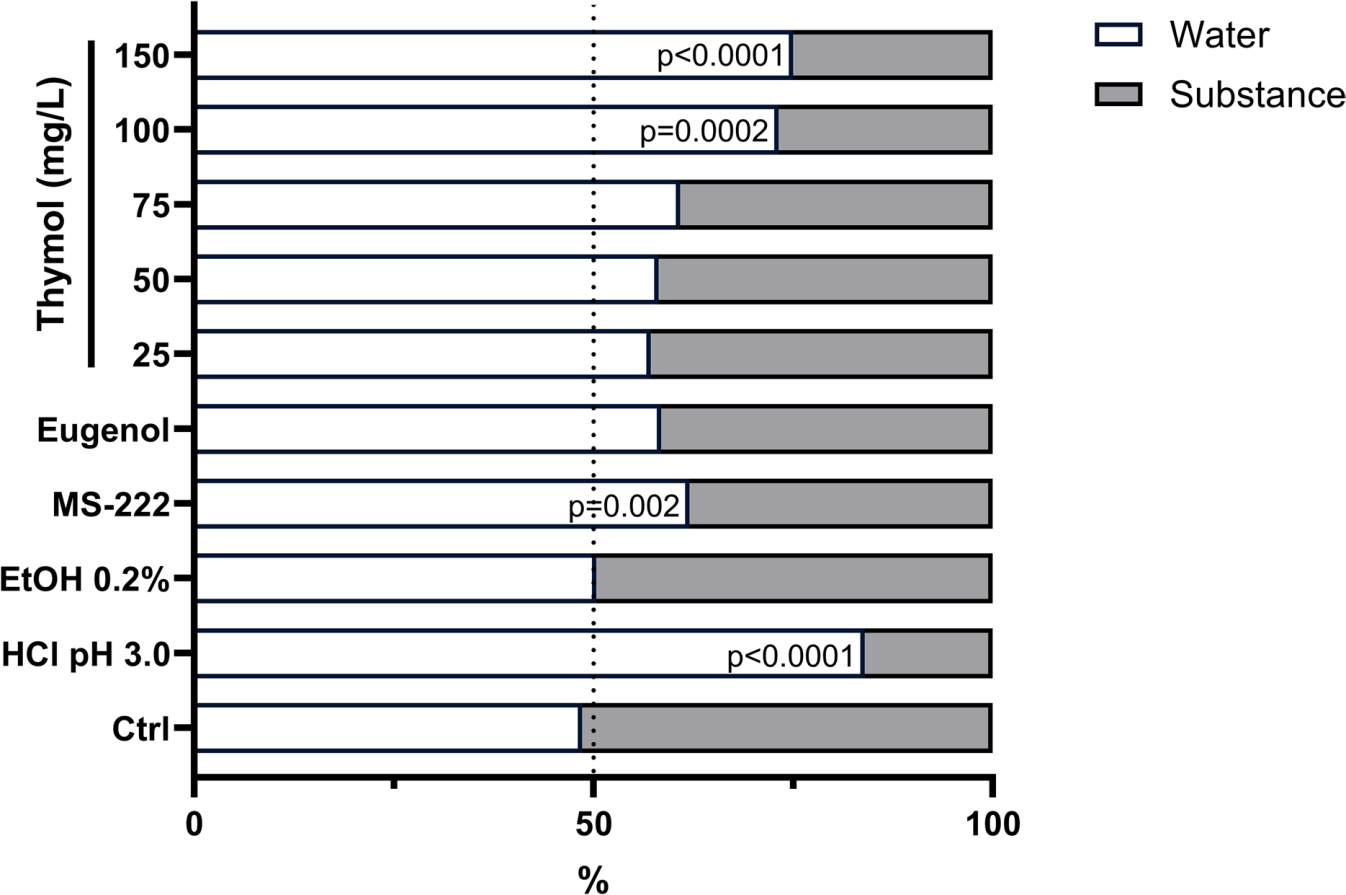
Percentage (%) of time zebrafish spent in the treated lane versus the water lane during the substance exposure period. Data are expressed as the mean ± standard deviation (SD) from 10 independent observations and p-values are shown after each bar

The results from the preference test showing the time spent in the clean or conditioned zone are shown in Fig. 5, while the source data can be found in Supplementary Table 3. No preference was observed in both the control and ethanol groups (p = 0.703 and p = 0.912, respectively). However, in the positive control group (HCl pH 3.0), zebrafish exhibited a distinct aversive reaction, showing a tendency to spend more time in the clean zone than in the conditioned zone (p < 0.0001). Upon the introduction of thymol into the system, animals exhibited an aversive reaction to concentrations of 100 and 150 mg/L (p < 0.05). There was no notable preference observed for the lowest concentrations of thymol, nor for the tested eugenol concentration. Conversely, upon the introduction of MS-222 into the system, animals displayed a significant preference for remaining on the clean side of the system (p = 0.002).

### Thymol exposure decreases cortisol levels

**Fig. 6:**
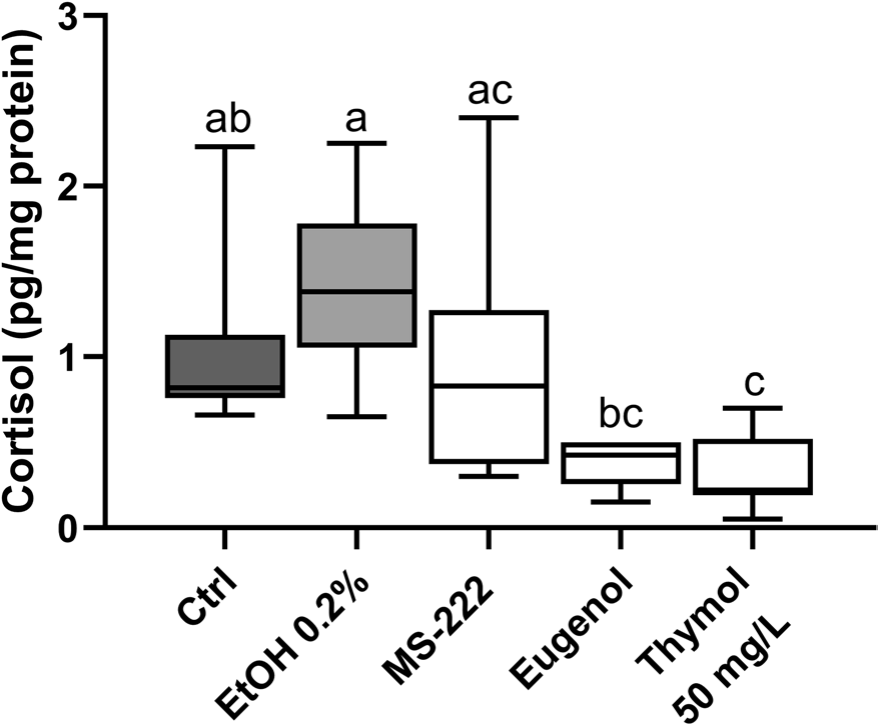
Cortisol levels in mucus of adult zebrafish following 10-min thymol anaesthesia. The values are presented as median and interquartile ranges from ten independent replicate exposures and were analysed by the Kruskall-Wallis test followed by Dunn’s post hoc test. Different letters indicate significant differences between groups (p < 0.05)

Figure 6 presents the results of cortisol level analysis following an extended period of anaesthesia (10 min) at a thymol concentration of 50 mg/L, while the original data can be found in Supplementary Table 4. A significant decrease in cortisol levels was observed for thymol anaesthetised animals compared to the control groups (p < 0.05), albeit no differences were observed, compared to the MS-222 and eugenol groups. The eugenol anaesthetised fish also showed diminished cortisol levels relative to the ethanol-exposed group (p = 0.001).

## Discussion

MS-222 and eugenol, commonly used anaesthetics in fish research and aquaculture, are associated with certain side effects (Carter et al. 2011; Barbas et al. 2021; Ayala-Soldado et al. 2023). The search for alternative methods to anesthetise fish has led to the consideration of monoterpenes as potential candidates, with studies showing efficacy in inducing anaesthesia in fish while often being considered safer and less toxic compared to traditional anaesthetics (Félix et al. 2023). In this study, thymol, a natural monoterpene compound commonly found in thyme, oregano, and other aromatic plants was tested for zebrafish anaesthesia, a model with potential application in aquaculture (Ulloa et al. 2014; Lee-Estevez et al. 2018; Jorgensen 2020; Piferrer and Ribas 2020). The findings indicate that thymol exhibits anaesthetic effects that vary with concentration although resembling those of eugenol and MS-222. Physiological and behavioural reactions were comparable across the anaesthetics, even though animals exposed to MS-222 exhibited aversive behaviour, a response only seen with high concentrations of thymol (exceeding 100 mg/L). In addition, following a prolonged exposure of 10 min to thymol, cortisol levels decreased.

Due to the differing reactions to various anaesthetics (Readman et al. 2017), it is essential to assess different concentrations when testing new fish species. Typically, the optimal level of anaesthesia through immersion baths is achieved within 5 to 10 min, ensuring minimal mortality rates and enabling a prompt recovery to their natural state, usually within 10 min (Neiffer and Stamper 2009). According to this, in the current study, thymol was examined as an alternative anaesthetic for zebrafish, with the effective anaesthetic concentration established between 50 and 75 mg/L. Although no study can be found in the literature regarding the anaesthetic profile of thymol in adult zebrafish, and despite the potential interspecies sensitivity to anaesthetics (Readman et al. 2017), similar concentrations have been described in adult *Garra rufa* (Aydın and Orhan 2021). However, higher concentrations (> 200 mg/L) are needed to anaesthetise 96-h zebrafish larvae (Vieira et al. 2024). This was expected as age is one of the factors that can affect fish's response to anaesthesia (Sneddon 2012) and a higher resistance of zebrafish larvae to anaesthetics when compared to adults has been shown (Matthews and Varga 2012). Still, similar range concentrations (50–100 mg/L) have been described as effective to anaesthetise different juvenile species such as *Cyprinus carpio* (Yousefi et al. 2018), *Oreochromis niloticus* (Yousefi et al. 2022), *Rhamdia quelen* (Bianchini et al. 2017) and *Colossoma macropomum* (Boaventura et al. 2022). Overall, these findings underscore the imperative for a more methodical examination of toxicodynamic and toxicokinetic effects during fish anaesthesia assessment which can enhance the safety and efficacy of anaesthetic procedures. Notwithstanding, the anaesthetic profile of thymol at the given concentrations was within the range determined for MS-222 and eugenol, as well as documented in the literature (Grush et al. 2004; Collymore et al. 2014; Jorge et al. 2021; Baesso et al. 2024), among others, further supporting its use as anaesthetic. Curiously, the lowest concentration (25 mg/L) of thymol failed to induce deep anaesthesia within the stipulated time (10 min) despite displaying potential sedative effects, as observed by the induction of stage A2. Still, similar effects have been observed in other species using equal concentrations (Bianchini et al. 2017; Yousefi et al. 2018). In a broader context, these findings could hold significance for procedures requiring fish sedation, such as transportation. Beyond these findings, no significant correlation could be established between thymol concentrations and induction time while a positive correlation with recovery time was perceived which was inconsistent to what has been described in the literature (Bianchini et al. 2017; Yousefi et al. 2018, 2022; Aydın and Orhan 2021; Boaventura et al. 2022). This apparent contradictory outcome may be due to species differences in anaesthetic sensitivity and resistance to state transitions as previously described (McKinstry-Wu et al. 2019). Yet, only further evaluation of the neuronal dynamics under thymol anaesthesia will clarify this phenomenon. Despite this, thymol anaesthesia induced a depression in the cardiorespiratory system even at the lowest concentration. Overall, a disruption of this system has been previously suggested in other aquatic species while also causing prolongation of the QT-interval (Aydın and Orhan 2021). Although not assessed in the present study, long Q-T intervals predispose individuals to lethal cardiac arrhythmias (Leong et al. 2010). It's important to point out that cardiac arrhythmias are characteristic of fish anaesthesia (Soldatov 2021), characterised as temporary and reversible for various monoterpenes (Félix et al. 2023). In addition, a report suggests that thymol can affect fish respiration and/or oxygenation (Mirzargar et al. 2022), which is consistent with the observed decrease in ventilatory frequency. It is important to note that when the highest concentration was administered (200 mg/L), the ventilatory frequency nearly ceased, which may explain the highest mortalities observed in the same group. Overall, while low concentrations (< 100 mg/L) seem to be safe for fish anaesthesia, high concentrations of thymol should be avoided, at least for this species, due to the risk of hypoxia. However, further studies should assess detailed cardiac function (e.g. electrocardiogram recordings or QT interval measurements) as well as evaluate potential histological changes to gain a more comprehensive understanding of the systemic effects of thymol anaesthesia.

In addition to its known GABAergic activity in fish (Bianchini et al. 2017), thymol’s interaction with this receptor can influence behaviour and neural dynamics (Weir et al. 2017). In this study, while MS-222 produced minimal behavioural changes during induction, consistent with previous findings in zebrafish (Nordgreen et al. 2014), the lowest thymol concentration (50 mg/L) did not cause significant behavioural alterations, while higher concentrations induced short bursts of hyperactivity followed by reduced activity. This biphasic response has been reported with other anaesthetic, both natural and synthetic (Ross and Ross 2009; Pereira-da-Silva et al. 2016; Uehara et al. 2019), and is likely attributed to the irritating nature of the anaesthetic agents (Readman et al. 2013). Nevertheless, the highest thymol doses elicited rapid swimming and escape-like movements, suggestive of an aversive or panic-like response, possibly an attempt to reduce branchial exposure. In fact, a previous study has shown signs of avoidance behaviour (e.g. increased distance, swimming speed and mobility percentage) in thymol-anesthetized fish (Aydın and Orhan 2021) which were associated with a deterioration of the fish welfare level. Still, and although further investigation is guaranteed, these discrepancies may stem from species-specific sensitivity to thymol (Readman et al. 2013). Notably, no aversive responses were observed at concentrations ≤ 75 mg/L, whereas higher doses triggered clear avoidance. Although literature on thymol aversion in fish is scarce, essential oils rich in monoterpenes have generally not been shown to cause attraction or aversion (Junior et al. 2018). Additionally, neither ethanol nor eugenol induced aversion, while MS-222 altered lane preference in line with earlier zebrafish and other fish studies (Readman et al. 2013, 2017). While the underlying mechanism in zebrafish requires further clarification, the current findings suggest the potential use of concentrations ranging from 50–75 mg/L of thymol as a possible alternative for anaesthesia in adult zebrafish.

Additionally, fish tend to exhibit aversive behaviours when exposed to stressful stimuli such as a predator, poor water quality, or drugs, among others (Le et al. 2019a; Ferreira et al. 2022). This activates the release of cortisol from the adrenal-like interrenal cell which is a known indicator of stress in fish (Wendelaar Bonga 1997). In the present study, mucus cortisol levels significantly decreased in 10-min thymol-exposed fish, compared to the control groups. Although similar approaches were not found in the existing literature, a study described an increase in cortisol levels following short anaesthesia with thymol (30–80 mg/L) in juvenile *Oreochromis niloticus* (Yousefi et al. 2022). This contradictory finding can be explained by the divergent time course of cortisol response in fish at different ages (Barcellos et al. 2012; Koakoski et al. 2012). While the peak of cortisol is attained within 5-min of the stressor exposure for juveniles, there is a 1-h increasing latency until the peak in adult stages. Yet, although statistical differences are also observed within 5-min of the stressor exposure, the referred studies were conducted in *Rhamdia quelen,* and both inter and intraspecies differences in response to stress and environmental perturbations have been reported (Winberg et al. 2016). Furthermore, the reports on anaesthesia-induced cortisol responses in fish are conflicting, with synthetic anaesthetics increasing cortisol levels while natural anaesthetics decreasing or maintaining its levels (Small 2003; Cunha et al. 2010; Zahl et al. 2010). Therefore, a more extensive investigation is necessary to fully understand the relationship between thymol anaesthesia and the activation of the HPI axis in fish. For instance, thymol’s interaction with GABA_A_ receptors (Bianchini et al. 2017), along its potential effects on endocrine and immune modulation (Alagawany et al. 2021) may contribute to the suppression of cortisol release. Regardless of this, and similar to what was observed in the present study, no significant cortisol changes were reported between thymol and eugenol during short-term anaesthesia (5 min) in *Cyprinus carpio* (Yousefi et al. 2018) at concentrations similar to those used here. Overall, these findings support the potential of thymol to be applied as an alternative anaesthetic for adult zebrafish.

Overall, the anaesthetic profile of thymol is comparable to that of commonly used anaesthetics, with no behavioural aversion or significant cortisol changes observed at effective concentrations. The present findings indicate that thymol is effective at 50–75 mg/L, providing reliable anaesthesia in zebrafish with minimal adverse effects. In contrast, higher concentrations (> 100 mg/L) were associated with physiological stress and reduced welfare and should therefore be used with caution. Based on these results, a working concentration of 50–75 mg/L is recommended for adult zebrafish. To expand the applicability of thymol in fish anaesthesia, future studies should investigate its physiological effects more deeply, including haemato-biochemical parameters, electrophysiological responses, pharmacokinetics, neuronal impacts, and long-term outcomes across different species and life stages.

## Supplementary Information

Below is the link to the electronic supplementary material.

Supplementary file1 (DOCX 27 KB)

Supplementary file2 (AVI 8386 KB)

## Data Availability

No datasets were generated or analysed during the current study.
