# Supplementary material for: Evaluation of thymol application for anaesthesia of adult zebrafish
Source: Fish Physiol Biochem. 2025 Sep 1;51(5):155. doi: 10.1007/s10695-025-01574-z (PMC12402031; doi:10.1007/s10695-025-01574-z)
Supplement: Supplementary file 1 — Supplementary file1 (DOCX 27 KB) [file 10695_2025_1574_MOESM1_ESM.docx]

|  | **Concentration (mg/L)** | **Time (seconds)** | | **Heart rate (bpm)** | **Ventilatory frequency (min)** | **Time (seconds)** | | **Fish mortality (%)** |
| --- | --- | --- | --- | --- | --- | --- | --- | --- |
|  |  | **A2** | **A3** |  |  | **R1** | **R2** |  |
|  | 0 | - | - | 205 [149-254] ^a^ | 261 [239-315] ^a^ | - | - | 0 |
| EtOH | 0.2 % | - | - | 180 [150-238] ^a^ | 254 [240-283] ^a^ | - | - | 0 |
| MS-222 | 150 | 126 [86-161] ^a^ | 363 [293-446] ^ab^ | 131 [113-153] ^ab^ | 84 [54-130] ^ab^ | 46 [33-102] ^a^ | 128 [79-167] ^a^ | 0 |
| Eugenol | 80 | 18 [16-22] ^bc^ | 157 [132-180] ^bd^ | 75 [63-98] ^bc^ | 31 [8-55] ^bc^ | 104 [73-158] ^ab^ | 163 [123-238] ^ab^ | 0 |
| Thymol | 25 | 45 [35-50] ^ab^ | > 600 | 104 [96-134] ^ac^ | 34 [32-69] ^ac^ | - | - | 0 |
|  | 50 | 11 [9-21] ^c^ | 330 [191-424] ^abc^ | 89 [66-99] ^bc^ | 53 [38-61] ^ac^ | 257 [165-477] ^bc^ | 357 [261-504] ^bd^ | 0 |
|  | 75 | 17 [10-35] ^bc^ | 280 [194-413] ^abc^ | 90 [63-116] ^bc^ | 24 [12-33] ^bc^ | 466 [290-572] ^c^ | 530 [339-601] ^cd^ | 0 |
|  | 100 | 8 [6-13] ^c^ | 109 [75-128] ^d^ | 111 [86-134] ^ac^ | 70 [34-87] ^ab^ | 180 [126-263] ^ac^ | 197 [153-279] ^abc^ | 10 |
|  | 150 | 10 [6-13] ^c^ | 131 [92-224] ^cd^ | 87 [79-129] ^ac^ | 34 [21-56] ^bc^ | 189 [172-396] ^bc^ | 234 [192-397] ^ad^ | 20 |
|  | 200 | 13 [9-14] ^c^ | 473 [392-555] ^a^ | 65 [50-84] ^c^ | 4 [0-9] ^c^ | 1181 [1033-1308] ^c^ | 1256 [1157-1455] ^d^ | 90 |
| *Statistical test* | | X^2^(7)=55.05 | X^2^(6)=46.64 | X^2^(9)=56.91 | X^2^(9)=70.59 | X^2^(5)=39.55 | X^2^(5)=34.96 |  |
| *p-value* | | <0.0001 | <0.0001 | <0.0001 | <0.0001 | <0.0001 | <0.0001 |  |

**Table S1 - Latencies to induction and recovery stages, heart-rate, ventilatory frequency and mortality in adult zebrafish exposed to different concentrations of thymol.**

Data from ten independent observations expressed as median and interquartile range. Statistical analysis was performed using the Kruskal-Wallis test followed by Dunn’s post hoc comparison test. Different superscripts denote significant differences between groups in same column (p<0.05).

**Table S2 - Behavioural response of zebrafish to different concentrations of thymol until the loss of equilibrium (up to 180 seconds of analysis, stage A2).**

|  | **Concentration (mg/L)** | **Total distance (m)** | | **Average speed (cm/s)** | | **Meandering (º/m)** | | **Time freezing (s)** |
| --- | --- | --- | --- | --- | --- | --- | --- | --- |
|  | 0 | 1.87 [1.52-2.59] ^a^ | | 10.4 [8.45-14.4] ^a^ | | 12.8 [11.4-16.8] ^ab^ | | 1.45 [0.58-16.3] ^a^ |
| EtOH | 0.2 % | 1.74 [1.26-3.19] ^ab^ | | 9.70 [6.95-17.8] ^a^ | | 13.0 [8.70-15.4] ^ab^ | | 1.80 [0.20-17.0] ^a^ |
| MS-222 | 150 | 1.18 [0.80-1.42] ^ac^ | | 6.55 [4.40-7.93] ^ab^ | | 16.2 [13.3-19.2] ^a^ | | 15.1 [3.98-19.7] ^ab^ |
| Eugenol | 80 | 0.61 [0.50-0.80] ^c^ | | 3.40 [2.80-4.48] ^b^ | | 14.0 [10.6-16.2] ^ab^ | | 29.8 [29.2-33.8] ^c^ |
| Thymol | 25 | 1.28 [0.84-2.20] ^ac^ | | 7.10 [4.65-12.3] ^ab^ | | 9.50 [9.00-11.9] ^ab^ | | 22.2 [9.10-32.4] ^ac^ |
|  | 50 | 0.99 [0.73-1.66] ^ac^ | | 5.50 [4.05-9.23] ^ab^ | | 10.3 [8.75-12.4] ^b^ | | 26.3 [24.0-36.7] ^bc^ |
|  | 75 | 1.37 [0.80-1.52] ^ac^ | | 7.60 [4.45-8.43] ^ab^ | | 10.7 [9.20-12.0] ^b^ | | 22.4 [12.3-31.8] ^ac^ |
|  | 100 | 0.72 [0.69-1.05] ^c^ | | 4.00 [3.88-5.70] ^b^ | | 12.3 [9.18-13.7] ^ab^ | | 31.2 [28.8-33.5] ^c^ |
|  | 150 | 0.89 [0.79-0.95] ^bc^ | | 5.05 [4.50-5.88] ^ab^ | | 10.2 [8.45-13.6] ^b^ | | 30.0 [24.2-33.3] ^bc^ |
|  | 200 | 0.95 [0.91-1.34] ^ac^ | | 5.30 [5.10-7.45] ^ab^ | | 11.1 [9.70-11.3] ^ab^ | | 24.8 [17.3-27.5] ^ac^ |
| *Statistical test* | | X^2^(9)=42.21 | X^2^(9)=42.61 | | X^2^(9)=25.65 | | X^2^(9)=46.76 | |
| *p-value* | | <0.0001 | <0.0001 | | 0.002 | | <0.0001 | |

Data from ten independent observations expressed as median and interquartile range. Statistical analysis was performed using the Kruskal-Wallis test followed by Dunn’s post hoc comparison test. Different superscripts denote significant differences between groups in same column (p<0.05).

**Table S3 - Aversive responses of zebrafish to different concentrations of thymol.**

|  | **Concentration (mg/L)** | **Time in water (s)** | **Time in substance (s)** | **Statistical test** | **p-value** |
| --- | --- | --- | --- | --- | --- |
|  | 0 | 48.6 ± 16.1 | 51.4 ± 16.1 | t=0.387 | 0.703 |
| HCl | pH 3.0 | 84.0 ± 6.97 | 16.0 ± 6.97 | t=21.81 | <0.0001 |
| EtOH | 0.2 % | 50.4 ± 17.2 | 49.6 ± 17.2 | t=0.106 | 0.912 |
| MS-222 | 150 | 62.1 ± 14.6 | 37.9 ± 14.6 | t=3.709 | 0.002 |
| Eugenol | 80 | 58.5 ± 24.4 | 41.8 ± 24.4 | t=1.562 | 0.136 |
| Thymol | 25 | 57.2 ± 20.3 | 42.8 ± 20.3 | t=1.514 | 0.149 |
|  | 50 | 58.2 ± 18.1 | 41.8 ± 18.1 | t=1.919 | 0.073 |
|  | 75 | 60.9 ± 25.6 | 39.1 ± 25.6 | t=1.905 | 0.073 |
|  | 100 | 73.2 ± 22.1 | 26.8 ± 22.1 | t=4.695 | 0.0002 |
|  | 150 | 75.0 ± 19.3 | 25.0 ± 19.3 | t=5.794 | <0.0001 |

Values are expressed as mean ± standard deviation of 10 independent animals. Statistical analysis was made using the t-test in comparison to control group values (p < 0.05).

**Table S4 – Cortisol levels in adult zebrafish exposed to different anaesthetics for 10 min period.**

|  | **Concentration (mg/L)** | **Cortisol**  **(pg/mg protein)** | |
| --- | --- | --- | --- |
|  | 0 | 0.82 [0.76-1.13] ^ab^ | |
| EtOH | 0.2 % | 1.38 [1.06-1.78] ^a^ | |
| MS-222 | 150 | 0.83 [0.37-1.28] ^ac^ | |
| Eugenol | 50 | 0.43 [0.26-0.50] ^bc^ | |
| Thymol | 50 | 0.22 [0.19-0.52] ^c^ | |
| *Statistical test* | | X^2^(4)=21.65 |  |
| *p-value* | | 0.0002 |  |

Data from ten independent replicates expressed as median and interquartile range. Statistical analysis was performed using the Kruskal-Wallis test followed by Dunn’s post hoc comparison test. Different superscripts denote significant differences between groups in same column (p<0.05).
